# Supplementary material for: Systems‐level phosphoproteomics reveals calcium/calmodulin‐dependent protein kinase kinase 2‐driven kinase signaling underlying malignant phenotypes in gastric cancer
Source: J Cell Commun Signal. 2026 Oct 27;20(4):e70112. doi: 10.1002/ccs3.70112 (PMC13712449; doi:10.1002/ccs3.70112)
Supplement: Supplementary file 1 [file CCS3-20-e70112-s003.docx]

**Systems level phosphoproteomics reveals CAMKK2 driven kinase signaling underlying malignant phenotypes in gastric cancer**

Mohd. Altaf Najar^1*^, T. S. Keshava Prasad^1,2^, Prashant Kumar Modi^1*^

^1^Center for Systems Biology and Molecular Medicine, Yenepoya (Deemed to be University), Mangalore 575018, India

^2^ Nitte (Deemed to be University), Center for Omics & Systems Medicine (C-OSM), Department of Biochemistry, K.S. Hegde Medical Academy (KSHEMA), Mangalore - 575018, Karnataka, India

***Correspondence:**

Mohd Altaf Najar. Ph.D.,

Assistant Professor

Center for Systems Biology and Molecular Medicine

Yenepoya Research Centre

Yenepoya (Deemed to be University)

Mangalore 575018, India

Email: mohammadanajar@yenepoya.edu.in

Mobile: +91-9797271421

Prashant Kumar Modi, Ph.D.

Associate Professor

Center for Systems Biology and Molecular Medicine

Yenepoya Research Centre

Yenepoya (Deemed to be University)

Mangalore 575018, India

Email: prashantmodi@yenepoya.edu.in

Mobile: +91-9873662911

**Authors**

Mohd. Altaf Najar	mohammadanajar@yenepoya.edu.in

T. S. Keshava Prasad	Keshava.prasad@nitte.edu.in

Prashant Kumar Modi	prashantmodi@yenepoya.edu.in

**Abstract**

Gastric cancer is driven by aberrant kinase signaling that promotes uncontrolled proliferation and malignant progression. Calcium/calmodulin-dependent protein kinase kinase 2 (CAMKK2) is overexpressed in gastric cancer; however, the global phosphorylation networks downstream of CAMKK2 remain incompletely defined. In this study, we investigated the functional and signaling consequences of CAMKK2 inhibition in gastric cancer cells using an integrated phenotypic and quantitative phosphoproteomics approach. Pharmacological inhibition of CAMKK2 using STO-609 in AGS cells significantly suppressed proliferation, clonogenic growth, migration, and invasion, and induced defects in nuclear morphology indicative of impaired cell cycle progression. Tandem mass tag (TMT) based phosphoproteomic profiling identified over 10,500 phosphopeptides and revealed extensive phosphoproteome remodeling following CAMKK2 inhibition, characterized predominantly by hypophosphorylation of proteins involved in nuclear signaling, RNA processing, and cell cycle regulation. Kinase substrate enrichment and motif analyses demonstrated coordinated attenuation of CDK, MAPK, and mitotic kinase-associated signaling pathways, with convergence on E2F regulated transcriptional programs. Collectively, these findings establish CAMKK2 as a central regulator of kinase signaling networks that sustain proliferative and malignant phenotypes in gastric cancer and highlight CAMKK2 inhibition as a potential therapeutic strategy.

**Keywords**

CAMKK2, gastric cancer, phosphoproteomics, kinase signaling, cell cycle regulation, MAPK signaling, RNA metabolism, systems biology

**Abbreviations**

CAMKK2, Calcium/calmodulin-dependent protein kinase kinase; GC, Gastric cancer; TMT, Tandem mass tag; LC-MS/MS, Liquid chromatography tandem mass spectrometry; GO, Gene Ontology; KSEA, Kinase substrate enrichment analysis; MAPK, Mitogen activated protein kinase; CDK, Cyclin-dependent kinase; ERK, Extracellular signal-regulated kinase; AKT, Protein kinase B; ATM, Ataxia telangiectasia mutated; ATR, Ataxia telangiectasia and Rad3-related; PRKDC, Protein kinase DNA-activated catalytic subunit; E2F, E2 factor transcription factor; PCA, Principal component analysis; FDR, False discovery rate; PTM, Post-translational modification; Fe-NTA, Ferric nitrilotriacetic acid; TiO₂, Titanium dioxide; ACN, Acetonitrile;
TFA, Trifluoroacetic acid; TEABC, Triethylammonium bicarbonate; SEM, Standard error of the mean; SDS-PAGE, Sodium dodecyl sulfate–polyacrylamide gel electrophoresis; DHB, 2,5-Dihydroxybenzoic acid; X2K, eXpression2Kinases.

**Results**

**Supplementary Figure S1: Protein protein interaction network clustering analysis of CAMKK2-regulated phosphoproteins:** Protein–protein interaction network generated using differentially phosphorylated proteins following CAMKK2 inhibition in AGS gastric cancer cells. Network clustering analysis identified distinct functional modules associated with (A) cell cycle and nuclear envelope organization, (B) ribosomal proteins, and (C) RNA splicing machinery. Node color intensity represents clustering score.

**Supplementary Figure S2: Hierarchical Gene Ontology enrichment analysis of CAMKK2-regulated phosphoproteins:** Hierarchical Gene Ontology (GO) enrichment tree showing significantly enriched biological processes associated with CAMKK2-regulated phosphoproteins. Major enriched pathways include RNA processing, mRNA splicing, chromatin organization, regulation of gene expression, metabolic processes, and cellular regulatory pathways. Node size corresponds to gene count, and node color intensity represents enrichment significance (−log10 p-value).

**Supplementary Figure S3: Kinase-ranking analysis of CAMKK2-regulated phosphoproteome:** Kinase-ranking analyses using MeanRank and TopRank scoring approaches identifying significantly enriched kinase signaling networks associated with CAMKK2 inhibition. Prominent kinase families include SRPK, CDK, CSNK2A1, AURKB, CLK, and AKT signaling pathways. Bar plots represent integrated kinase enrichment scores and overlapping substrate proteins.
