# Supplementary material for: Systems‐level phosphoproteomics reveals calcium/calmodulin‐dependent protein kinase kinase 2‐driven kinase signaling underlying malignant phenotypes in gastric cancer
Source: J Cell Commun Signal. 2026 Oct 27;20(4):e70112. doi: 10.1002/ccs3.70112 (PMC13712449; doi:10.1002/ccs3.70112)
Supplement: Supplementary file 2 [file CCS3-20-e70112-s005.docx]

**Supplementary Table S1.** Complete list of identified phosphopeptides and phosphorylation sites quantified in AGS gastric cancer cells following vehicle or STO-609 treatment. The table includes protein accession numbers, gene names, peptide sequences, phosphorylation site(s), localization probability, normalized phosphopeptide abundances, and corresponding quantitative measurements used for downstream phosphoproteomic analysis.

**Supplementary Table S2.** Differentially regulated phosphopeptides identified following CAMKK2 inhibition by STO-609. The table includes significantly altered phosphopeptides with corresponding protein and phosphosite information, protein abundance-normalized phosphorylation ratios, fold changes, statistical significance (p-values and adjusted p-values/FDR, where applicable), and the direction of regulation.

**Supplementary Table S3.** Statistically significant **serine-centered phosphorylation motifs** identified from all significantly hypophosphorylated phosphosites following CAMKK2 inhibition. Motif enrichment analysis was performed using **MoMo**. The table includes motif sequences, regular expression patterns, enrichment scores, foreground and background matches, unadjusted p-values, and multiple testing-adjusted p-values (FDR).

**Supplementary Table S4.** Statistically significant **threonine-centered phosphorylation motifs** identified from all significantly hypophosphorylated phosphosites following CAMKK2 inhibition. Motif enrichment analysis was performed using **MoMo**. The table includes motif sequences, regular expression patterns, enrichment scores, foreground and background matches, unadjusted p-values, and multiple testing-adjusted p-values (FDR).
